# Hsa_circ_0000105 promotes nasopharyngeal carcinoma malignancy by miR-541-3p/S100A11 axis

**DOI:** 10.1016/j.clinsp.2025.100577

**Published:** 2025-01-28

**Authors:** LuXing Peng, Jian Qin, DeFeng Qing, XinJun Huang, XiuRong Huang

**Affiliations:** a Department of Clinical Oncology Center, Radiotherapy Ward 3, The People's Hospital of Guangxi Zhuang Autonomous Region, Nanning City, Guangxi Zhuang Autonomous Region, China; b Department of Clinical Oncology Center, Radiotherapy Ward 2, The People's Hospital of Guangxi Zhuang Autonomous Region, Nanning City, Guangxi Zhuang Autonomous Region, China

**Keywords:** RNA, circular, Nasopharyngeal neoplasms, MicroRNAs, S100A11 protein, Epithelial-mesenchymal transition

## Abstract

**Objective:**

This study was to investigate whether hsa_circ_0000105 is involved in the process of regulating Nasopharyngeal Carcinoma (NPC) biological behaviors and to reveal the molecular mechanism.

**Methods:**

NPC tissues and normal tissues were collected, and NPC cell lines and normal control cell lines were obtained. hsa_circ_0000105/miR-541-3p/S100A11 was evaluated by RT-qPCR or Western blot. CCK-8 assay, EdU assay, Transwell assay, wound healing assay, flow cytometry, and Western blot were employed to evaluate the biological behaviors of NPC cells. Dual luciferase reporter assay and RIP assay were applied to evaluate the direct targeting relationship of hsa_circ_0000105/miR-541-3p/S100A11. A tumor xenotransplantation assay was implemented to evaluate the effect of hsa_circ_0000105 on NPC tumors.

**Results:**

hsa_circ_0000105 and S100A11 were highly expressed in NPC, while miR-541-3p was lowly expressed. hsa_circ_0000105 competitively adsorbed miR-541-3p and mediated S100A11 expression. Silencing or upregulation of hsa_circ_0000105 restricts and induces malignant behavior in HNE2 cells, respectively. The impact of hsa_circ_0000105 silencing on malignant behaviors of HNE2 cells was blocked by miR-541-3p downregulation, while that of hsa_circ_0000105 upregulation was attenuated by S100A11 inhibition.

**Conclusion:**

hsa_circ_0000105 acts as a carcinogenic factor in NPC and promotes NPC malignancy by miR-541-3p/S100A11 axis.

## Highlights

• hsa_circ_0000105 modulates malignancy of NPC cells.
• hsa_circ_0000105 influences the malignant behavior of NPC via downstream miR-541-3p.
• hsa_circ_0000105 positively regulates S100A11 to accelerate the malignant behavior of NPC.
• hsa_circ_0000105 inhibits NPC tumor growth *in vivo*.

## Introduction

Cancer of the nasopharynx, known as Nasopharyngeal Carcinoma (NPC), has a highly geographical distribution, with the highest incidence in Southern China and Southeast Asia.^1^ It is common for NPC patients to experience cervical lymph node metastases at diagnosis since early symptoms aren't obvious or specific.^2^^,^^3^ Despite radiotherapy and chemotherapy's effectiveness against early NPC, easy metastasis and high rates of early diagnosis diminish its effectiveness.^4^^,^^5^ NPC metastasis is a challenging scientific problem requiring a study of the molecular mechanisms involved.

NPC involves multiple factors, including molecular regulatory mechanisms such as genes and noncoding RNA (ncRNA).^6^^,^^7^ Circular RNA (circRNA) is a special type of ncRNA with a circular structure that coordinates NPC biological processes.^8^ For example, circRNAs have been identified to be.^9^, ^10^, ^11^, ^12^ In high-throughput sequencing, the authors focused on a circRNA named hsa_circ_0000105 that is highly expressed in NPC.^13^ However, it is not clear whether it regulates the biological behavior of NPC.

In this study, the methods of molecular biology, cell biology, and bioinformatics were integrated to fully understand the function of has_circ_0000105 in NPC and its downstream molecular mechanism. This study may provide new directions for developing more effective treatment strategies and improving patient survival. Through this work, the authors aim to provide useful insights into circRNA functions and applications in the field of NPC research.

## Materials and methods

### Clinical samples

Tumor tissue and normal tissue (> 3 cm from tumor tissue) were collected from 47 patients who underwent nasopharyngeal tumor biopsy and were pathologically diagnosed with NPC between September 2018 and March 2022. Patient information is shown in Table 1. This study obtained approval from the Ethics Committee of The People's Hospital of Guangxi Zhuang Autonomous Region (Ethical number: 201710GX03). All subjects have provided informed consent. All animal and clinical studies were conducted following ARRIVE guidelines.

**Table 1:** Relationship between HSP90B1 and clinicopathological characteristics of NPC patients.

Characteristic	Cases	The expression of hsa_circ_0000105		p
	*n* = 47	Low (*n* = 23)	High (*n* = 24)	
**Gender**				0.4711
**Male**	31	14	17	
**Female**	16	9	7	
**Age(year)**				0.4537
≤ 60	21	9	12	
> 60	26	14	12	
**Tumor size**				0.6763
< 3 cm	28	13	15	
≥ 3 cm	19	10	9	
TNM stage				0.4231
I/II	17	7	10	
II/IV	30	16	14	
Lymph node metastasis				0.0022
Positive	23	6	17	
Negative	24	17	7	
Distal metastasis				0.1718
Positive	28	16	12	
Negative	19	7	12	

### Real-time reverse transcriptase-polymerase chain reaction (RT-qPCR)

Total RNA was extracted from cells and tissues using TRIzol reagent (Invitrogen, MA, USA). MiRNAs were reverse transcribed and amplified using the All-in-One™ miRNA RT-qPCR Detection Kit (GeneCopoeia, Inc., USA) on the 7500 Fast Real-Time PCR System (Applied Biosystems, Thermo Fisher Scientific, USA). CircRNAs and mRNAs were quantified using the TB Green Premix EX Taq™ (Takara, Tokyo, Japan), with glyceraldehyde-3-phosphate dehydrogenase and U6 as internal controls. The relative gene expression was calculated using the 2^−∆∆Ct^ method. Primer sequences are provided in Table 2.

**Table 2:** RT-qPCR primer sequence.

Factors	Primer sequence (5′–3′)
hsa_circ_0000105	Forward: 5′-GCTGGGCAATGTGTTTGTCA-3′
	Reverse: 5′-TTCTTGCCCGACATAAGCAGA-3′
miR-541-3p	Forward: 5′-GTGGTGGGCACAGAATC-3′
	Reverse: 5′-TGGTGTCGTGGAGTCG-3′
S100A11	Forward: 5′-GGTGTCCTTGACCGCATGAT-3′
	Reverse: 5′-CAAGCCATAGCTAGGCCACC-3′
GAPDH	Forward: 5′-CACCCACTCCTCCACCTTTG-3′
	Reverse: 5′-CCACCACCCTGTTGCTGTAG-3′
U6	Forward: 5′-CTCGCTTCGGCAGCACA-3′
	Reverse: 5′-AACGCTTCACGAATTTGCGT-3′

### NPC cell lines

NPC cell lines (CNE2, HONE1, HNE2, HK1) and the immortalized nasopharyngeal epithelial cell line NP69 were obtained from ATCC (USA) and cultured in Roswell Park Memorial Institute (RPMI)−1640 supplemented with 10 % Fetal Bovine Serum (FBS) (Gibco, Grand Island, NY, USA) at 37 °C in a humidified 5 % CO_2_ atmosphere. All cell lines were authenticated using short tandem repeat profiling and tested mycoplasma-free.

### Actinomycin D and RNase R assays

RNA stability was assessed by treating with RNase R enzyme (Thermo Fisher, NY, USA) and actinomycin D (Sigma, Missouri, USA). Total RNA extracted from HNE2 cells was incubated with or without RNase R (20 U/μL) at 37 °C for 30 min followed by deactivation at 70 °C for 10 min for subsequent RT-qPCR analysis. Actinomycin D was added to HNE2 cells at a final concentration of 1 μg/mL at 0 h, 6 h, 12 h, and 24 h, and RNA was harvested for RT-qPCR.

### Northern blot

Total RNA and RNA treated with RNase R were analyzed using the NorthernMax® Kit (Invitrogen). Briefly, samples were run on a 1 % formaldehyde-polyacrylamide-urea gel, transferred to a positively charged Hybond N^+^ membrane (Amersham), and cross-linked by UV illumination. The membrane was hybridized with 3′-digoxigenin-labeled hsa_circ_0000105 probes (Axl-Bio, Guangzhou, China) overnight at 50 °C.

### Cell transfection

Cells were transiently transfected with si-0000105, si-S100 Calcium Binding Protein A11 (S100A11), pcDNA 3.1-0000105, miR-541-3p mimic, miR-541-3p inhibitor, and their respective negative controls using Lipofectamine 3000 (Life Technologies, NY, USA). Transfection efficiency was evaluated by RT-qPCR and Western blot 48 h post-transfection.

### Cell counting kit (CCK)-8 assay

Cell proliferation was assessed using the CCK-8 kit (Dojindo Laboratories, Kumamoto, Japan). Briefly, approximately 1 × 10^3^ HNE2 cells were seeded per well in a 96-well plate and incubated with 10 µL of CCK-8 solution at 0, 24, 48, and 72 h. The optical density at 450 nm was measured using a microplate reader after 2 h of incubation.

### 5-ethynyl-2′deoxyuridine (EdU) assay

Cell proliferation was further evaluated using the Fluor488 Click-iT EdU Imaging Kit (keyGEN Bio TECH, Nanjing, China). HNE2 cells were incubated with 50 µM EdU for 2 h at 37 °C, fixed, and then incubated in Click-iT reaction buffer for 30 min before staining with Hoechst to visualize nuclei. Images were captured using the EVOS FL Auto Imaging System (Life Technologies Corp., Bothell, WA, USA) and analyzed using ImageJ software.

### Transwell assay

The invasive capabilities of HNE2 cells were assessed using a Transwell chamber (Corning CoStar, USA). Cells were seeded in the upper chamber in serum-free RPMI-1640 medium at a density of 2 × 10^4^ cells/well. The lower chamber contained medium supplemented with 10 % FBS. After 12 h of incubation, cells were fixed with 4 % paraformaldehyde and stained with 0.1 % crystal violet for visualization. Cells were counted and images were captured under a light microscope.

### Scratch assay

Cells were seeded in six-well plates. At 24 h post-transfection, when confluency was observed under a microscope, three standardized scratches were made per well using a sterile 20 µL pipette tip. Serum-free RPMI 1640 replaced the medium containing 10 % FBS. Scratch widths were photographed at 0 and 24 h using a phase-contrast microscope.

### Flow cytometry

Apoptosis was quantified using the Cell Apoptosis Assay Kit (Beyotime, Shanghai, China). Cells were collected and stained according to the manufacturer's instructions with Annexin V-Fluorescein Isothiocyanate (FITC) and Propidium Iodide (PI). Double staining was conducted by incubating with 5 µL of Annexin V-FITC and 5 µL of PI in the dark for 15 min. Apoptosis was measured using FACScan flow cytometry (Becton Dickinson, Mountain View, CA, USA).

### Western blot

Total proteins from tissues and cells were lysed using radioimmunoprecipitation assay buffer (Beyotime Biotechnology, Shanghai, China) containing a protease/phosphatase inhibitor mixture (Roche Applied Sciences, Mannheim, Germany), separated by 10 % sodium dodecyl sulfate-polyacrylamide gel electrophoresis, and transferred to a polyvinylidene fluoride membrane (Millipore, Billerica, MA, USA). The membrane was blocked with 5 % non-fat milk in Tris-Buffered Saline and Tween 20 at room temperature for 2 h and probed with primary antibodies overnight at 4 °C. After washing, horseradish peroxidase-conjugated secondary antibodies (CUSBIO, Wuhan, China) were incubated at room temperature for 2 h. Proteins were visualized using enhanced chemiluminescence reagents (Millipore, Billerica, MA, USA). Primary antibodies included E-cadherin, N-cadherin, Snail, Ki-67, and cleaved caspase-3.

### Nucleocytoplasmic separation assay

Nuclear and cytoplasmic fractions were isolated from cells using the PARIS Kit (Ambion, USA). Cells (1 × 10^7^) were mixed with 0.5 mL of cytoplasmic extraction buffer (Invent Biotechnologies, Inc.) in an ice-cold petri dish for 10 min. The lysate was transferred to a test tube, vigorously shaken for 30 s, and centrifuged at 12,000×*g* at 4 °C for 15 min. TRIzol was added to extract RNA, and the expressions of hsa_circ_0000105, miR-541-3p, U6, and 18S were detected by RT-qPCR.

### Dual luciferase reporter assay

hsa_circ_0000105 and S100A11 3′UTR sequences containing miR-541-3p541-3p binding sites were amplified and cloned into the pmirGLO vector (Promega, Madison, WI, USA) to generate Wild-Type (WT) reporters, namely 0000105-WT and S100A11-WT. Mutant Type (MUT) hsa_circ_0000105 and S100A11 3′UTR sequences were cloned into empty luciferase reporter vectors to obtain 0000105-MUT and S100A11-MUT. Subsequently, these vectors were co-transfected into HNE2 cells with miR-541-3p mimic and mimic NC using Lipofectamine 3000 (Life Technologies, NY, USA). The relative luciferase activity was measured using the Dual Luciferase Reporter Analysis System (Promega, Madison, WI, USA).

### Fluorescence *in situ* hybridization (FISH) experiment

FISH detection was performed using CY3-labelled hsa_circ_0000105 probes and 488-labelled locked nucleic acid miR-541-3p probes.^14^ The probes were designed and synthesized by RiboBio (Guangzhou, China). HNE2 cells were permeabilized with 4 % paraformaldehyde + Triton X-100. Subsequently, cells were incubated with hsa_circ_0000105 or miR-541-3p probes overnight. Nuclei were stained with 4,6-diamidino-2-phenylindole (Life Technologies, Carlsbad, CA, USA) at room temperature for 10 min. Fluorescence images were acquired using a fluorescence microscope (BX51TRF; Olympus, Tokyo, Japan).

### RNA immunoprecipitation (RIP) experiment

Ago2-RIP was performed using the EZ-Magna Kit (Millipore, Bedford, MA, USA). Briefly, cell lysates were prepared with RIP lysis buffer and centrifuged. The cell lysates were then incubated with magnetic beads pre-coated with human AGO2 antibody in RIP buffer. The protein was digested with proteinase K, and RNA was subsequently purified. RNA relative expression was assessed by RT-qPCR.

### Tumor xenotransplantation experiment

Ten nude mice (BALB/c, specific pathogen-free grade, male, 4‒5 weeks of age) were sourced from Beijing Vital River Laboratory Animal Technology Co., Ltd. (Beijing, China) and housed under specific pathogen-free conditions. All experiments were conducted following the animal care and use guidelines of The People's Hospital of Guangxi Zhuang Autonomous Region and received ethical approval (Ethical number: 201809GX122). Mice were subcutaneously injected in the flank with HNE2 cells (4 × 10^7^ cells/200 µL) either with stable knockdown or without knockdown of hsa_circ_0000105. Tumor size was measured weekly with calipers, and the volume was calculated as (length) × (width)^2^/2. Mice were euthanized, and subcutaneous tumors were excised, weighed, and processed for subsequent Immunohistochemistry (IHC) staining^15^ and Western blot analysis.

### Data analysis

Data are presented as mean ± Standard Deviation (SD). All data underwent a minimum of three biological replicates. The Shapiro-Wilk test was used to assess normality. The Student's *t*-test was appropriate for comparing two groups, one-way analysis of variance was used for multiple group comparisons, and the Tukey HSD test was employed for post-hoc pairwise comparisons. A p-value of < 0.05 was considered statistically significant. The Chi-Square test was used to assess the correlation between hsa_circ_0000105 expression and clinical-pathological features of NPC patients.

## Results

### Aberrantly high expression of hsa_circ_0000105 in NPC

**Fig. 1:**
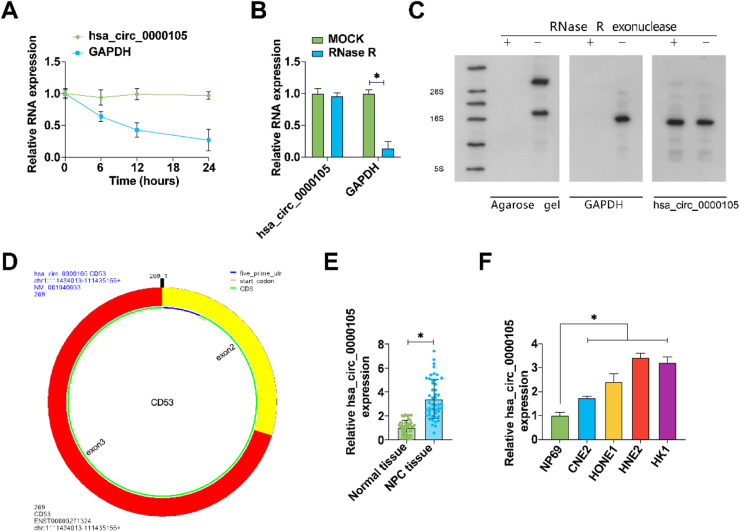
Aberrant overexpression of hsa_circ_0000105 in NPC. (A) Evaluation of the circular structure of hsa_circ_0000105 via actinomycin D assay; (B) RNase R assay confirming the resistance of hsa_circ_0000105 to degradation; (C) Northern blot analysis verifying the circular structure of hsa_circ_0000105; (D) Bioinformatics analysis mapping hsa_circ_0000105 on chromosome-1; (E) RT-qPCR analysis of hsa_circ_0000105 expression in NPC and adjacent normal tissues; (F) Expression analysis of hsa_circ_0000105 in NPC cell lines and the NP69 cell line. Data are presented as mean ± SD (*n* = 3); * *p* < 0.05.

Initially, the circular structure of hsa_circ_0000105 was evaluated. Resistance to RNase R digestion and actinomycin d-induced stability confirmed its circular RNA characteristics (Fig. 1A‒B). These observations were further substantiated by Northern blot analysis (Fig. 1C). Bioinformatics analysis using circPrimer 2.0 located hsa_circ_0000105 on chr1: 111434013–111435155, encompassing exons 2 and 3 of the CD53 gene (Fig. 1D). Quantitative RT-PCR analysis revealed significantly elevated levels of hsa_circ_0000105 in NPC tissues compared to adjacent non-tumoral tissues (Fig. 1E), and this upregulation was also observed across various NPC cell lines relative to the immortalized nasopharyngeal epithelial cell line NP69 (Fig. 1F). The association of hsa_circ_0000105 expression with clinical-pathological features of NPC patients, including lymph node metastasis, was statistically significant (Table 1), underscoring its potential role in NPC pathogenesis.

### Regulation of NPC proliferation, invasion, migration, epithelial-to-mesenchymal transition (EMT), and apoptosis by hsa_circ_0000105

**Fig. 2:**
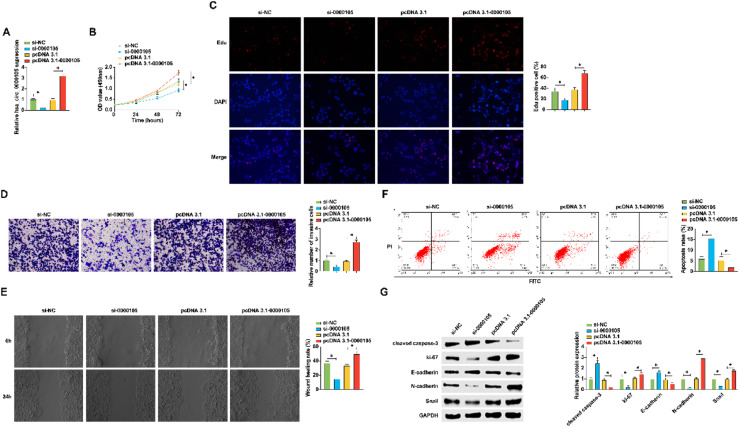
hsa_circ_0000105 regulates NPC proliferation, invasion, migration, EMT, and apoptosis. (A) RT-qPCR assessment of hsa_circ_0000105 expression following transfection with siRNA or overexpression vectors in HNE2 cells; (B) CCK-8 assay for proliferation rate; (C) Edu staining for DNA synthesis; (D) Transwell assay for invasion capability; (E) Wound healing assay for migration ability; (F) Flow cytometry for apoptosis rate; (G) Western blot analysis of proliferation, apoptosis, and EMT markers. Data are presented as mean ± SD (*n* = 3); * *p* < 0.05.

Following the initial observations, the authors modulated the expression of hsa_circ_0000105 in HNE2 cells using targeted siRNA and overexpression vectors (pcDNA 3.1). Successful modulation was confirmed by RT-qPCR (Fig. 2A). Subsequent assays including CCK-8 and Edu staining demonstrated that silencing hsa_circ_0000105 significantly reduced proliferation and DNA synthesis rates, whereas its overexpression enhanced these parameters (Fig. 2B, C). Transwell assays revealed that hsa_circ_0000105 knockdown markedly inhibited cell invasion, while its overexpression promoted this capability (Fig. 2D). Wound healing assays assessing migration corroborated these findings, showing reduced migration upon knockdown and increased migration with overexpression (Fig. 2E). Flow cytometry analysis indicated that silencing hsa_circ_0000105 significantly increased apoptosis, contrary to the effects seen with overexpression (Fig. 2F). Western blot analysis of proliferation, apoptosis, and EMT markers further supported these observations; knockdown of hsa_circ_0000105 decreased the expression of Ki-67, N-cadherin, and Snail, and increased cleaved caspase-3 and E-cadherin levels, with overexpression having the opposite effect (Fig. 2G). These results suggest a pro-oncogenic role for hsa_circ_0000105 in NPC progression.

### Targeting miR-541-3p by hsa_circ_0000105

**Fig. 3:**
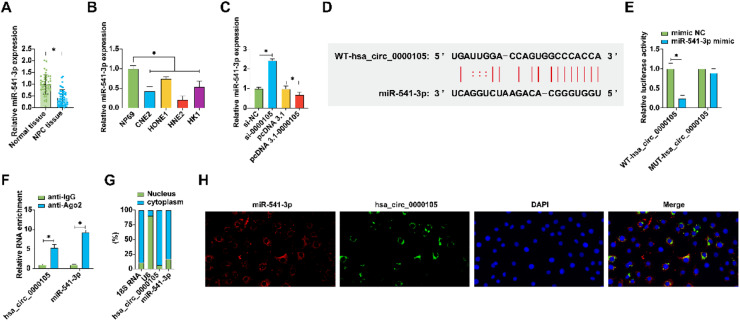
Has_circ_0000105 targets and sequesters miR-541-3p. (A) RT-qPCR evaluation of miR-541-3p expression in NPC tissues and adjacent non-tumor tissues; (B) Expression analysis of miR-541-3p in NPC cell lines and NP69 cells; (C) RT-qPCR quantification of miR-541-3p following modulation of has_circ_0000105 expression; (D) Schematic representation of the wild-type and mutant binding sites for has_circ_0000105 on the luciferase reporter vectors; (E) Dual-luciferase reporter assay validating the direct interaction between has_circ_0000105 and miR-541-3p; (F) RIP demonstrating the enrichment of has_circ_0000105 and miR-541-3p in the RISC complex; (G) Nuclear-cytoplasmic fractionation showing the subcellular localization of has_circ_0000105 and miR-541-3p; (H) FISH confirming the colocalization of has_circ_0000105 and miR-541-3p. Data are presented as mean ± SD (*n* = 3); * *p* < 0.05.

Further analysis focused on identifying potential downstream miRNAs regulated by hsa_circ_0000105. Among the candidates predicted by StarBase, miR-541-3p stood out due to its documented tumor-suppressive roles in various cancers.^16^^,^^17^ The authors found that miR-541-3p was upregulated in NPC tissues compared to adjacent non-tumor tissues (Fig. 3A) and was also more abundant in NPC cell lines than in the NP69 cell line (Fig. 3B). Manipulating hsa_circ_0000105 levels showed a reciprocal regulation of miR-541-3p, with knockdown enhancing and overexpression reducing miR-541-3p levels (Fig. 3C). Luciferase reporter assays using vectors containing either wild-type or mutant binding sites for hsa_circ_0000105 confirmed direct interaction with miR-541-3p (Fig. 3D, E). RIP experiments further substantiated the enrichment of hsa_circ_0000105 and miR-541-3p on Ago2 beads, supporting their interaction in the RISC complex (Fig. 3F). Subcellular fractionation and FISH experiments confirmed their cytoplasmic colocalization (Fig. 3G, H), highlighting the functional relevance of this interaction in cellular contexts.

### Regulatory influence of hsa_circ_0000105 on NPC malignant behavior via miR-541-3p expression

**Fig. 4:**
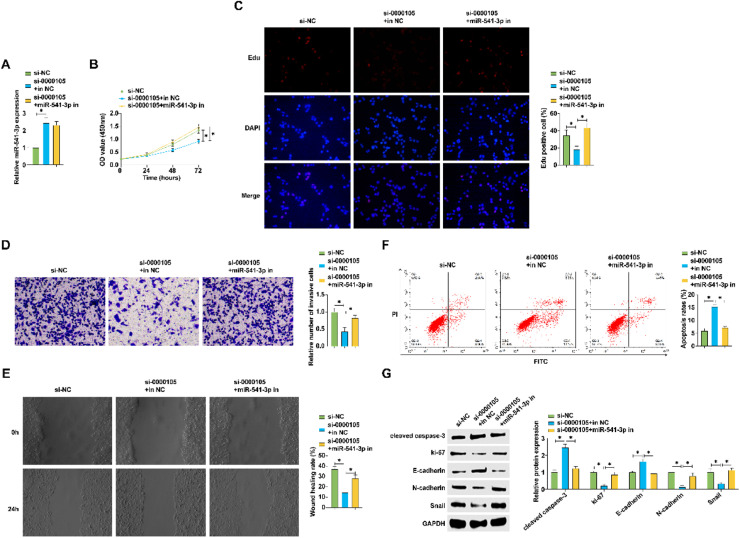
hsa_circ_0000105 modulates NPC malignancy through regulation of miR-541-3p expression. (A) RT-qPCR assessing the impact of hsa_circ_0000105 siRNA and miR-541-3p inhibitor on miR-541-3p levels in HNE2 cells; (B) CCK-8 assay for proliferation rate; (C) Edu staining quantifying proliferative cells; (D) Transwell assay for invasive cell count; (E) Wound healing assay assessing cellular migration; (F) Flow cytometry for apoptosis rate; (G) Western blot analysis of Ki-67, N-cadherin, Snail, cleaved caspase-3, and E-cadherin expression. Data represent mean ± SD (*n* = 3); * *p* < 0.05.

Further experiments utilized co-transfection of hsa_circ_0000105-targeting siRNA and a miR-541-3p inhibitor in HNE2 cells. The effects of si-0000105 on enhancing miR-541-3p expression were not reversed by the miR-541-3p inhibitor (Fig. 4A). Functional assays demonstrated that hsa_circ_0000105 knockdown decreased cell proliferation and invasion, effects which were mitigated by simultaneous knockdown of miR-541-3p, indicating functional interdependence (Fig. 4B‒D). Apoptosis assays revealed an increase in cell death upon hsa_circ_0000105 silencing, an effect attenuated by miR-541-3p inhibition (Fig. 4F). Protein expression analyses further showed that hsa_circ_0000105 knockdown reduced Ki-67, N-cadherin, and Snail levels, while increasing cleaved caspase-3 and E-cadherin, effects reversed by miR-541-3p silencing (Fig. 4G). These findings underscore the role of hsa_circ_0000105 in modulating NPC malignancy through miR-541-3p-dependent mechanisms.

### miR-541-3p downstream target gene S100A11

**Fig. 5:**
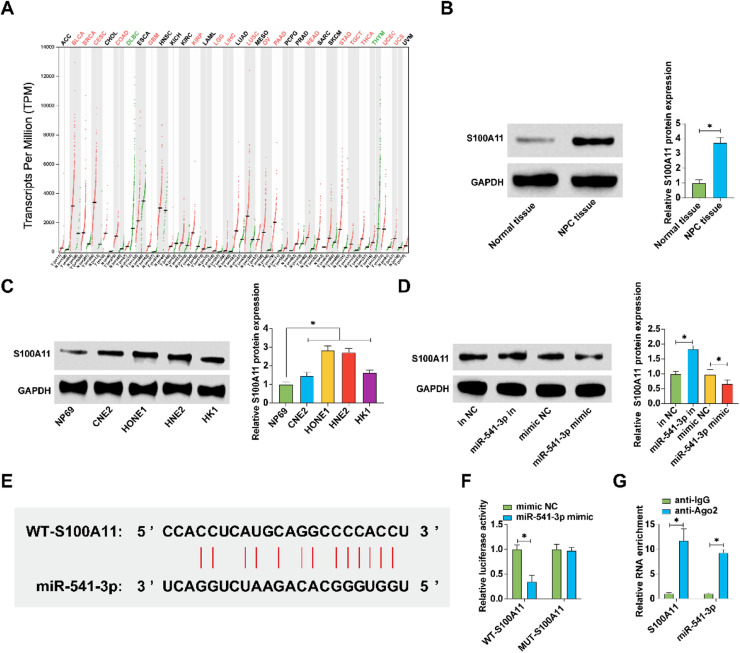
S100A11 as a downstream target gene of miR-541-3p. (A) GEPIA database analysis of S100A11 expression across various cancers; (B) Western blot analysis of S100A11 expression in NPC tissues and adjacent normal tissues; (C) Western blot quantifying S100A11 in NPC cell lines and the NP69 cell line; (D) Western blot analysis showing the impact of miR-541-3p modulation on S100A11 levels; (E) Schematic of S100A11 and miR-541-3p potential binding sites; (F) Dual-luciferase reporter assay confirming direct interaction; (G) RIP verifying the enrichment of S100A11 and miR-541-3p. Data represent mean ± SD (*n* = 3); * *p* < 0.05.

Subsequent investigations focused on the potential downstream targets of miR-541-3p. Among the predicted targets in the StarBase database, the gene S100A11 was noted for its elevated expression across various cancer tissues (Fig. 5A). Protein levels of S100A11, assessed by Western blot, were significantly higher in NPC tissues and cell lines compared to controls (Fig. 5B, C). Modulation of miR-541-3p levels inversely affected S100A11 protein levels, confirming a regulatory relationship (Fig. 5D). Luciferase assays with vectors carrying wild-type or mutant S100A11 binding sites demonstrated a direct interaction with miR-541-3p (Fig. 5F), which was further supported by RIP experiments showing enrichment of S100A11 and miR-541-3p on Ago2 beads (Fig. 5G). These results establish S100A11 as a direct downstream target of miR-541-3p.

### Positive regulation of S100A11 by hsa_circ_0000105 enhances NPC malignant behavior

**Fig. 6:**
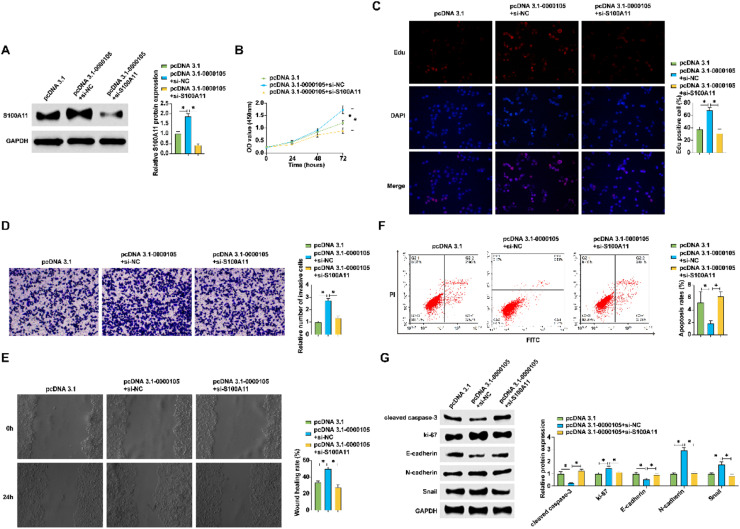
hsa_circ_0000105 positively regulates S100A11 expression to enhance NPC malignancy. (A) Western blot assessing the impact of hsa_circ_0000105 overexpression and S100A11 siRNA on S100A11 expression in HNE2 cells; (B) CCK-8 assay for proliferation rate; (C) Edu staining quantifying proliferative cells; (D) Transwell assay for invasive cell count; (E) Wound healing assay assessing cellular migration; (F) Flow cytometry for apoptosis rate; (G) Western blot analysis of Ki-67, N-cadherin, Snail, cleaved caspase-3, and E-cadherin expression. Data represent mean ± SD (*n* = 3); * *p* < 0.05.

Experiments involving co-transfection of a hsa_circ_0000105 overexpression vector and a siRNA targeting S100A11 in HNE2 cells showed that overexpression of hsa_circ_0000105 promoted S100A11 protein expression, an effect reversed by S100A11 silencing (Fig. 6A). This regulatory impact on S100A11 was correlated with changes in cellular proliferation, invasion, and apoptosis, with knockdown of S100A11 reversing the pro-tumorigenic effects of hsa_circ_0000105 overexpression (Fig. 6B‒F). Additionally, modulation of hsa_circ_0000105 altered the expression of key markers such as Ki-67, N-cadherin, and Snail, and these changes were mitigated by S100A11 knockdown (Fig. 6G). These findings indicate that hsa_circ_0000105 promotes NPC malignancy through upregulation of S100A11.

### In vivo suppression of NPC tumor growth by hsa_circ_0000105 knockdown

**Fig. 7:**
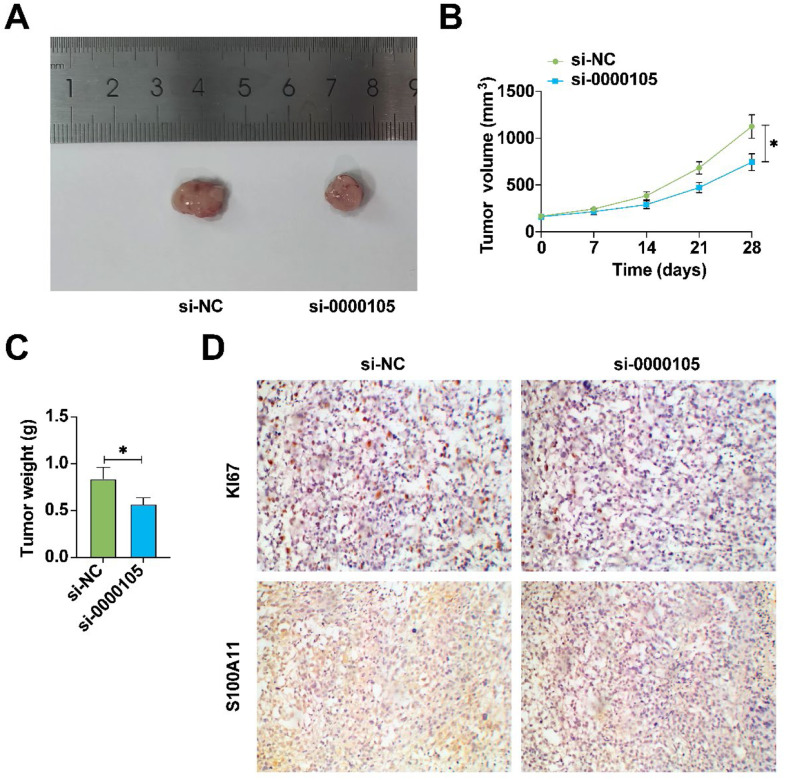
In vivo suppression of NPC tumor growth by hsa_circ_0000105. (A) Representative images of tumors; (B) Tumor volume; (C) Tumor weight; (D) Immunohistochemical staining for Ki-67 and S100A11 in tumor tissues. Data represent mean ± SD (*n* = 3); * *p* < 0.05.

In vivo experiments assessed the impact of hsa_circ_0000105 knockdown on NPC tumor growth. Results showed a significant reduction in tumor volume and weight following hsa_circ_0000105 silencing (Fig. 7A‒C). Additionally, immunohistochemistry revealed decreased expression of Ki-67 and S100A11 in the tumors (Fig. 7D), confirming the potential of hsa_circ_0000105 as a therapeutic target in NPC.

## Discussion

NPC clinically cannot be controlled due to local recurrences and distant metastases.^18^^,^^19^ Molecular mechanisms of NPC metastasis and proliferation should be explored, as well as active molecules involved. Although circRNA has shown a high degree of involvement in NPC proliferation and metastasis, more data are needed to construct the gene networks that circRNA regulates in NPC. This study found for the first time that hsa_circ_0000105, as a novel circRNA, promotes malignancy of NPC cells by miR-541-3p/S100A11 axis.

Circular RNAs are shown to regulate NPC development more and more often. For example, circPVT1 promotes NPC metastasis through a β-TrCP/c-Myc/SRSF1 axis.^8^ In addition, circCRI1, as a ceRNA, enhances NPC metastasis and chemotherapy resistance to docetaxel by up-regulating forkhead box Q1.^20^ In this work, upregulating hsa_circ_0000105 accelerated malignant behaviors of NPC cells This confirmed that hsa_circ_0000105 is an oncogene in NPC. Notably, a previous work finds that has_circ_0000105 is also an oncogene in liver cancer.^21^ In addition, has_circ_0000105 was highly associated with Lymph Node Metastasis (LNM) in NPC patients. It is worth noting that the LNM of NPC is the place of initial metastasis.^3^ In LNM in NPC, the malignant proliferation of cancer cells will lead to the enlargement of tumor volume and increase the chance of NPC entering the lymph node duct, while highly invasive and migratory NPC cells and EMT-developing NPC cells are more likely to enter the lymphatic duct.^22^^,^^23^ Therefore, has_circ_0000105′s promotion of NPC cell proliferation, invasion, migration, and EMT may be a cause of NPC LNM. It is worth noting that during the process of EMT in NPC cells, these cells are more likely to evade the monitoring of immune cells.^24^, ^25^, ^26^ Therefore, the effect of has_circ_0000105 on NPC immune microenvironment can be further explored in future studies.

Since circRNAs in the cytoplasm reduce miRNA inhibition on their target genes by binding and adsorption of miRNA^27^ the authors evaluated has_circ_0000105 and miR-541-3p subcellular localization. The authors found that has_circ_0000105 and miR-541-3p were both expressed in the cytoplasm of HNE2. These results indicate that has_circ_0000105 regulates downstream mRNA by adsorption of miR-541-3p. Previous studies have confirmed that miR-541-3p is a tumor suppressor, which includes prostate cancer, hepatocellular carcinoma, cervical cancer, etc.^16^^,^^28^^,^^29^ It was found that miR-541-3p, a tumor suppressor in NPC, inhibits the downstream gene S100A11 according to this study. A member of the S100 family of proteins, S100A11 interacts with calcium ions and is involved in biological processes that require calcium. S100A11 has been found to regulate the biological processes of various cancers and the survival prognosis of patients in the tumor microenvironment.^30^, ^31^, ^32^ The present work tested that S100A11 can also exert its cancer-promoting effect in NPC.

Firstly, while the study confirms the circular RNA characteristics and function of hsa_circ_0000105 in NPC using various in vitro and in vivo techniques, these findings are predominantly based on experimental manipulations in cell lines and may not fully represent the in vivo human disease environment. Additionally, the study heavily relies on RNA interference and overexpression systems, which might not precisely mimic natural expression levels and could lead to non-physiological cellular responses. The use of a single immortalized nasopharyngeal epithelial cell line as the normal control might not adequately represent the diversity of normal human tissues, potentially affecting the generalizability of the findings. Moreover, while the study implicates miR-541-3p and S100A11 as key mediators in hsa_circ_0000105-driven NPC pathogenesis, the mechanistic insights remain correlative, and causal relationships in a clinical context are yet to be established. This underscores the necessity for further studies integrating more comprehensive models and clinical samples to validate and extend these findings, thereby enhancing their translational potential for therapeutic interventions.

## Conclusion

In summary, the authors report a novel circRNA named has_circ_0000105 with powerful carcinogenic activity in NPC. NPC cells grow and become aggressive as a result of has_circ_0000105 adsorbing miR-541-3p and regulating S11A11 expression. These findings provide insights into the behavior of circRNAs in NPC and identify potential biomarkers and therapeutic targets.

## Funding

Not applicable.

## CRediT authorship contribution statement

**LuXing Peng:** Conceptualization, Investigation, Writing – original draft, Writing – review & editing. **Jian Qin:** Methodology, Investigation. **DeFeng Qing:** Methodology, Data curation. **XinJun Huang:** Formal analysis, Data curation. **XiuRong Huang:** Formal analysis, Data curation.

## Declaration of competing interest

The authors declare that they have no known competing financial interests or personal relationships that could have appeared to influence the work reported in this paper.
